# Association of human papillomavirus (HPV), p16, p53 and p63 expression with non-bilharzia-associated squamous cell carcinoma of the bladder and algorithm construction for histopathological grading prediction

**DOI:** 10.31744/einstein_journal/2023AO0109

**Published:** 2023-04-18

**Authors:** Patrícia Rocha Martins, Tálita Pollyanna Moreira dos Santos, Letícia Mattos Menezes, Astaruth Guimarães Froede, Matheus de Souza Gomes, Lucas Nogueira, Letícia da Conceição Braga, Laurence Rodrigues do Amaral, Paulo Guilherme de Oliveira Salles

**Affiliations:** 1 Núcleo de Ensino e Pesquisa Instituto Mário Penna Belo Horizonte MG Brazil Núcleo de Ensino e Pesquisa, Instituto Mário Penna, Belo Horizonte, MG, Brazil.; 2 Universidade Federal de Uberlândia Patos de Minas MG Brazil Universidade Federal de Uberlândia, Patos de Minas, MG, Brazil.; 3 Memorial Sloan Kettering Cancer Center New York NY USA Memorial Sloan Kettering Cancer Center, New York, NY, USA.; 4 Universidade Federal de Uberlândia Uberlândia MG Brazil Universidade Federal de Uberlândia, Uberlândia, MG, Brazil.

**Keywords:** Algorithms, Urinary bladder neoplasms, Human papillomavirus 16, Human papillomavirus type 53, Human papillomavirus 63, Papillomaviridae, Papillomavirus infections, Machine learning, Carcinoma, squamous cell

## Abstract

**Objective:**

To investigate the expression of human papillomavirus (HPV), p16, p53, and p63 in non-schistosomiasis-related squamous cell carcinoma of the bladder and to develop an accurate and automated tool to predict histological classification based on clinicopathological features.

**Methods:**

Twenty-eight patients with primary bladder pure squamous cell carcinoma who underwent cystectomy or transurethral resection of bladder tumor (TURBT) for bladder cancer between January 2011 and July 2017 were evaluated. Clinical data and follow-up information were obtained from medical records. Formalin-fixed, paraffin-embedded surgical specimens were used for immunohistochemical staining for p16, p53, and p63. Human papillomavirus detection was evaluated by PCR. Statistical analysis was performed, and statistical significance was set at p<0.05. Finally, decision trees were built to classify patients’ prognostic features. Leave-one-out cross-validation was used to test the generalizability of the model.

**Results:**

Neither direct HPV detection nor its indirect marker (p16 protein) was identified in most cases. The absence of p16 was correlated with less aggressive histological grading (p=0.040). The positive p16 staining detection found only in pT1 and pT2 cases in our sample suggests a possible role for this tumor suppressor protein in the initial stages of bladder squamous cell carcinoma. The decision trees constructed described the relationship between clinical features, such as hematuria/dysuria, the level of tumor invasion, HPV status, lymphovascular invasion, gender, age, compromised lymph nodes, and tumor degree differentiation, with high classification accuracy.

**Conclusion:**

The algorithm classifier approach established decision pathways for semi-automatic tumor histological classification, laying the foundation for tailored semi-automated decision support systems for pathologists.

**Figure:**
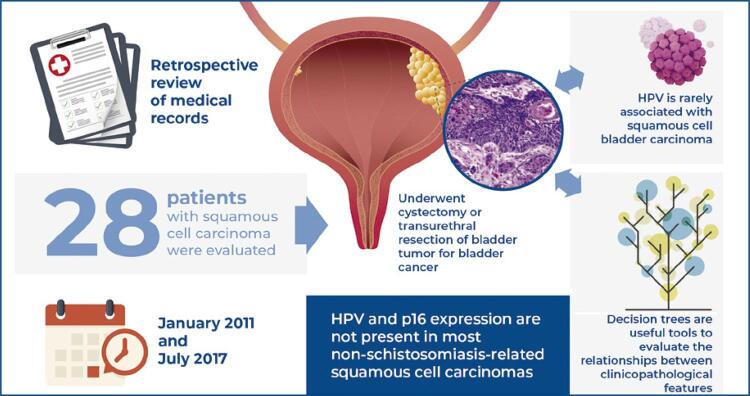

## INTRODUCTION

Bladder cancer is the tenth most prevalent malignancy worldwide, causing over 212,000 deaths annually.^(1)^ The number of new cases of bladder cancer estimated for Brazil, for each year for the 2020-2022 triennium, it will be 7,590 cases in men and 3,050 in women. These values correspond to an estimated risk of 7.23 new cases per 100,000 men and 2.80 per 100,000 women.^(2)^ The 5-year overall survival rate of urinary bladder cancer is 77%, and has not significantly changed over the past 30 years despite improvements in treatment.^(3)^

The spectrum of non-urothelial histological subtypes includes squamous cell carcinoma (SCC) (2%-5%), adenocarcinoma (0.5%-2%), and small cell carcinoma (<1%).^(4,5)^ The bladder’s SCC is divided into two categories based on its relationship with schistosomiasis: bilharzia-associated SCC and non-bilharzia-associated SCC. These types differ in pathogenesis, risk factors, epidemiology, and prognosis.^(6)^ Given the rare incidence of bladder SCC non-bilharzia-associated tumors in this study, we focused solely on this histologic subtype.

Tobacco exposure, repetitive urinary tract infections, and bladder irritants (such as stones) are well-known risk factors for bladder cancer development; notably, they share a common pathway for tumorigenesis, an environment of chronic inflammation. Recently, some types of human papillomavirus (HPV) have been associated with bladder SCC. However, no definitive relationship between HPV and SCC has been confirmed, and conflicting results have been reported.^(7)^ This viral infection is strongly carcinogenic and is associated with anogenital and head and neck neoplasia.^(8)^ Some studies have suggested that key cell cycle regulators, such as inactivation of the p16 pathway, can play a role as prognostic markers in SCC bladder. Inactivation of the p16 pathway, which controls the passage of cells from the G1 to the S phase, is a frequent event in all types of cancer. The p16 protein binds to cyclin-dependent kinases 4 and 6, thereby preventing phosphorylation of the retinoblastoma (RB) gene product. In HPV-related tumors, the E7 viral oncoprotein inactivates pRB, blocking its binding to the E2F transcription factor and promoting sustained cell cycle progression.^(9)^ These mechanisms lead to p16 overexpression in both the nucleus and cytoplasm and can be detected by immunohistochemistry. In non-HPV-related tumors, p16 overexpression occurs due to RB loss of function, whereas decreased p16 gene expression occurs due to deletion, mutations, or silencing. Regarding the prognostic value of p16 protein expression, reports are still conflicting between carcinomas from different primary sites and in the diverse histological subtypes of the same carcinoma. The E7 viral protein causes overexpression of the p16 protein, which has been consequently assumed to be an indirect marker of HPV-induced bladder cancer.^(10)^

In this context, machine learning (ML) has been applied in oncology to inform prognosis and to estimate the probabilities of cancer recurrence and progression, as well as survival.^(11)^ Our group and other researchers have used ML methods^(12-14)^ to improve biomedical translational research, which will greatly benefit the clinical management of patients with cancer.

Since HPV appears to have a causative role in the pathogenesis of bladder cancer and p16, p53, and p63 expression are reported to be involved in tumor development and progression, this study aimed to assess ML signature-related SCC of the bladder using clinicopathological outcome data.

## OBJECTIVE

To investigate HPV, p16, p53, and p63 expression in non-schistosomiasis-related squamous cell carcinoma of the bladder, and to develop an accurate and automated tool to predict its histological classification based on clinicopathological features.

## METHODS

### Case selection and pathological diagnosis

After obtaining approval from the Institutional Ethics Committee for human subjects (CAAE: 39670820.0.0000.5121; # 4.559.756), a retrospective chart review was conducted of all primary bladder cancer cases analyzed at the Pathology Division of the *Hospital Luxemburgo* at *Instituto Mário Penna* (Belo Horizonte, MG) between January 2011 and July 2017. Twenty-eight surgical specimens (cystectomy or transurethral resection) from patients with primary pure bladder SCC who underwent cystectomy or transurethral resection of bladder tumor (TURBT) for bladder cancer were identified. None of the patients had a known history of bladder cancer or predisposing factors, such as stones, infections, or schistosomiasis. A center review analysis of the whole slide sections was performed, and SCC specimens were classified according to the established parameters.^(15)^ Clinical data (sex, age, ethnic group, smoking history and alcohol consumption, tumor location, extension, T and N stages, weight loss, hematuria, dysuria, and pelvic pain) and follow-up information (clinical outcome and survival time) were obtained from medical records.

### Immunohistochemistry

All specimens consisted of paraffin-embedded blocks sectioned into 3-4µm slices and submitted to immunohistochemical staining of neoplastic cells. For tumor suppressor staining, mouse monoclonal antibodies directed against p53 (IgG2b; clone DO-7, Dako; Glostrup, Dania, Denmark) and P16INK (CINTec, Ventana; Tucson, Arizona, USA) at a 1:100 dilution, and p63 (clone 4A4, NeoMarkers; Fremont, CA, USA) at a 1:4.000 dilution were used. Prior to exposure to primary antibodies, whole tissue sections were subjected to antigen retrieval in a 10mM citrate buffer solution (pH 6.0) in a microwave for 20 minutes. To block endogenous tissue peroxide, the slides were immersed in 3% H_2_O_2_ solution at room temperature for 30 minutes and thoroughly washed with phosphate-buffered saline. Primary antibodies were incubated overnight at 4°C. After a 10 minutes-incubation period with the secondary antibody at room temperature, positive cells were marked with the streptavidin-peroxidase complex (Novolink polymer detection system, Novocastra, Newcastle), and the reactions were developed using a diaminobenzidine chromogen solution (0.03% of 3-3′-diaminobenzidine - SIGMA - containing 0.5% H_2_O_2_ in 0.01M phosphate-buffered saline, pH 7.4). Gill’s hematoxylin (Sigma-Aldrich) was used as a counterstain. Negative controls were used for each antibody. A pathologist evaluated the immunostained slides and determined the percentage of positive neoplastic cells.

### HPV detection

DNA extraction and HPV genotyping were performed using formalin-fixed and paraffin-embedded tissue specimens. Briefly, DNA was extracted by incubation with proteinase K (100μg/mL) at 55°C for 12-15 hours. DNA yields were spectrophotometrically quantified using a NanoVue spectrophotometer (GE Healthcare). To assess DNA quality, a PCR assay using five primer sets that amplified products from 100 to 600 base pairs was performed.^(16)^

HPV detection was first evaluated by PCR using the consensus primers GP5+ and GP6+. This method allows for the amplification of a broad spectrum of HPV genotypes. HPV-positive samples were detected for subtypes 6, 11, 16, 18, and 33 by multiplex PCR. To monitor DNA isolation efficiency, PCR amplification and genotyping procedures for each run were performed with positive and negative controls. An adequate absence of contamination was observed in the negative control of the HPV PCR assay.

### Statistical analysis

Data analysis was performed using GraphPad PRISM 5 (GraphPad Software Inc., San Diego, CA). Categorical variables were expressed as absolute numbers. Fisher’s exact test was used to evaluate the associations between two groups when there were fewer than five observations per cell. Statistical significance was set at p<0.05.

### Development and training of bladder SCC classifier algorithms

Decision trees were generated to select root attributes with higher accuracy to classify the subgroups. Thus, the J48 method, which is an implementation of C4.5, was applied. The J48 method was present in the WEKA software (Waikato Environment for Knowledge Analysis, version 3.6.11, University of Waikato, New Zealand). To test and validate the accuracy, robustness, and generalizability of the generated decision trees, the leave-one-out cross-validation method (LOOCV) was applied. LOOCV is a special case of cross-validation, in which the number of folds equals the number of instances in the dataset. Thus, the generated decision trees are tested for each instance of the dataset, using all other instances as a training set and the selected instance as a single-item test set. The final LOOCV result was the average of the errors found.

## RESULTS

### Demographic characterization and pathological data

**Figure 1:**
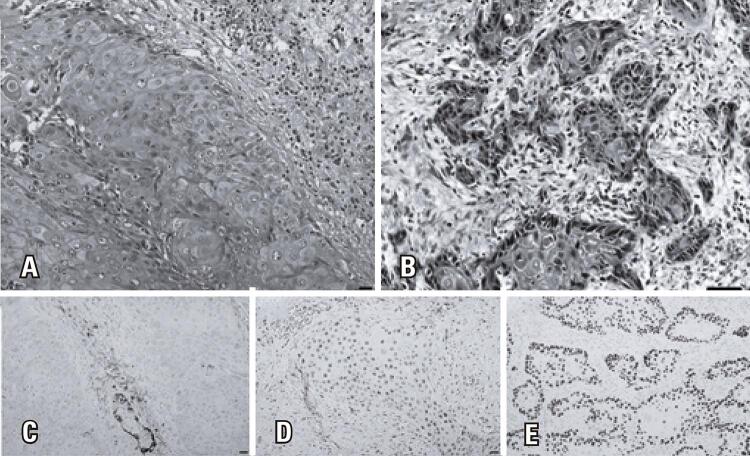
Hematoxylin-eosin-stained and immunohistochemistry sections from patients with squamous cell carcinoma of bladder. (A) extensive infiltration by tumor cells moderately differentiated. The cells are pleomorphic/atypical, large, and polygonal, with abundant eosinophilic cytoplasm and a central nucleus with evident an nucleolus (200x amplification); (B) malignant neoplasia composed of blocks of polygonal cells, which demonstrates foci of keratinization, keratin pearls and intercellular bridges in squamous cell carcinoma well differentiated (HE 400X); (C) p16 immunoreactive focally positive (200x amplification); (D) p53 focally immunoreactive (200x amplification); (E) p63 positivity widely distributed in all tumor areas (100x amplification). Scale bar 10μm

Patient demographics, clinical features, and initial pathological data are presented in table 1. The mean age at diagnosis was 65.53±9.96 (range 31-100) years, and the female-to-male ratio was 1:1. None of the patients had a history of *Schistosoma* infection or other medical conditions related to chronic inflammation of the genitourinary tract. Smoking history was present in of 12/28 (42.85%) patients. Histologically, all cases met the criteria for SCC, as seen in the hematoxylin-eosin-stained sections (Figure 1), and were reviewed and confirmed by a pathologist (PGOS). Most tumors showed pT2 invasion (60.71%) (Figure 1A). The percentage of moderately differentiated tumors was 71.42% (Figure 1B). None of the patients had previously received chemotherapy or radiotherapy.

**Table 1:** Patient demographic and clinical characteristics

Clinicopathological features	Findings n (%)
Gender	
Female	14 (50.0)
Male	14 (50.0)
Surgical procedure	
Cystectomy	10 (35.71)
Partial cystectomy	2 (7.14)
TURBT	16 (57.14)
Weight loss	
Yes	8 (28.57)
No	20 (71.43)
Hematuria	
Yes	21 (75.0)
No	7 (25.0)
Dysuria	
Yes	14 (50.0)
No	14 (50.0)
Pelvic pain	
Yes	16 (57.14)
No	12 (42.86)
Hypertension	
Yes	18 (64.29)
No	10 (35.71)
Smoking history	
Yes	12 (42.85)
No	11 (39.28)
Without information	5 (17.85)
Degree of differentiation (n=28)	
Well differentiated	8 (28.57)
Moderately differentiated	20 (71.42)
Poorly differentiated	0 (0.0)
Level of invasion (n=28)	
pT1	2 (7.14)
pT2	17 (60.71)
pT3	5 (17.85)
pT4	4 (14.28)
Lymphovascular invasion (n=28)	
Absence	20 (71.42)
Presence	8 (28.57)
Perineural invasion (n=28)	
Absence	14 (50.0)
Presence	14 (50.0)
Compromised lymph nodes (n=12)	
pN0	12 (42.85)
pN1/pN2	2 (7.14)
Without lymph nodes	14 (50.0)

TURBT: transurethral resection of bladder tumor.

### HPV, p16, p53 and p63 status

Regarding HPV analysis, the predominant proportion of cases was HPV negative (26/28; 92.85%) for either low- or high-risk HPV genotypes. Virus genotype determination was not possible, and p16 was focally (<5%) expressed in only one of the two HPV-positive cases.

Immunohistochemistry was performed to evaluate p16, p53, and p63 expression (Figure 1C, 1D, and 1E, respectively). As shown in table 2, most cases were p16 negative (20/28, 71.42%), p53 positive (22/28, 78.57%), and p63 positive (28/28, 100%). Among p16 positive cases, six out of eight were focally positive. There was no association between p16 staining and HPV status (p=0.49, two-sided Fisher’s exact test). An additional analysis of these cases was conducted, as 71.42% of patients tested negative for p16. When considering the combined group (pT1 + pT2) an association between the absence of p16 staining and less aggressive tumor invasion was found (p=0.040, Fisher’s exact test, two-sided). There was no association between p53 or p63 staining and HPV status (p=0.71, two-sided Fisher’s exact test). A subset of seven patients with both p16 and p53 positivity was also evaluated. When we compared this subset of patients with both p16 and p53 positivity staining, the patients with both positivity were older (on average). Regarding p63 positivity, no association with immunohistochemical markers or clinicopathological features was detected.

**Table 2:** Immunohistochemical characteristics by human papillomavirus status

Prognostic factor	HPV DNA status	
	Positive	Negative
p16		
Positive	0	2
Focally positive	1	5
Negative	1	19
p53		
Positive	0	18
Focally positive	2	4
Negative	0	6
p63		
Positive	2	26
Negative	0	0

HPV: human papillomavirus.

Most cases were ≥ pT2 stage disease and tended to have aggressive features, with 20/28 (71.42%) cases graded as moderately differentiated. However, most patients (12/28, 42.85%) were classified as pN0. Additionally, 8/28 (28.57%) cases had lymphovascular invasion and 14/14 (50.0%) cases had perineural invasion.

### Decision tree-based classifiers

**Figure 2:**
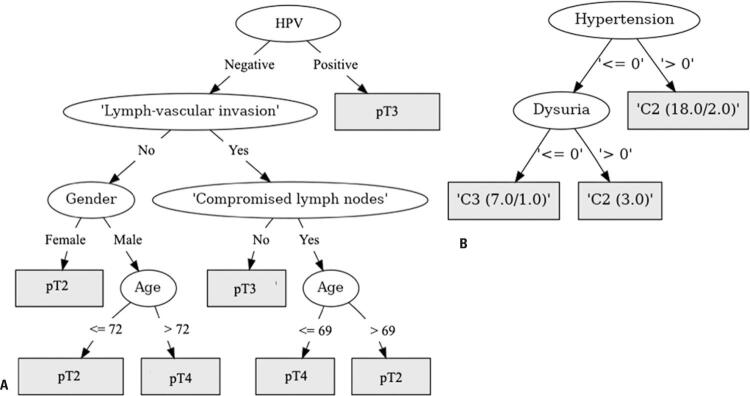
Classification tree structure and its performance in determining tumor differentiation degree (A) and level of invasion (B) in squamous cell carcinoma of the bladder. The algorithm showed the clinical data necessary to bladder squamous cell carcinoma classification

The classifier algorithm confirmed the prognostic value of the evaluated clinical and pathological features. Decision trees constructed for SCC of the bladder subgroups indentified the participants’ clinical features and HPV status as the major attributes for characterizing tumor differentiation degree (Figure 2A) and level of invasion (Figure 2B) with a global accuracy of 89.3% and 92,6%, respectively. When we evaluated the dataset using the LOOCV validation method, the algorithm correctly classified 23/28 samples for tumor differentiation degree (82.1% accuracy) and 17/28 samples (60.7% accuracy) for the level of invasion.

## DISCUSSION

The role of HPV infection in bladder carcinogenesis remains controversial.^(7,16,17)^ The virus is rarely associated with squamous cell bladder carcinoma and is not likely to have a significant effect on its genesis, as in cervical cancer.^(18,19)^ In our study, we were able to determine HPV positivity status only in two cases, which supports previous literature reports that demonstrate no etiologic role of HPV in the carcinogenesis of bladder SCC.^(19)^ Further corroborating our data, some investigations on bladder cancer have shown low or negative HPV-DNA detection by PCR or qPCR.^(10,20)^ Different studies have demonstrated the absence or low detection of HPV in urothelial carcinoma or SCC of the bladder.^(21-24)^ Although controversial, some studies have demonstrated that HPV infection is associated with the development of bladder carcinoma.^(17,22,25,26)^ However, a diverse experimental design approach might have an impact on the controversial results obtained by different studies regarding HPV infection in the carcinogenesis of bladder SCC. The sample source for genetic material acquisition is an important factor in HPV DNA detection. Ideally, fresh frozen tissue samples are the best source for HPV detection. Nevertheless, the current diagnostic method is formalin-fixed paraffin-embedded biopsy specimens, which may provide low levels of HPV DNA and compromise the diagnosis of viral infection.^(16)^ In our study, all genetic material used was acquired from formalin-fixed paraffin-embedded specimens, which might have affected HPV detection.

The p16 gene product plays a relevant role in tumor suppression by inhibiting the key cell cycle-related proteins CDK4/CDK6. As a result, alterations in p16 may lead to abnormal or malignant cell proliferation and acceleration of neoplasia development. Therefore, elucidation of the correlation between p16 low expression and clinicopathological features may provide new insights for early SCC diagnosis, treatment, and prognosis. In this context, our findings are consistent with the involvement of p16 in the tumor progression of bladder SCC, as evidenced by its negative immunostaining association with the early stages of tumor invasion (pT1 and pT2).

The other tumor suppressor proteins evaluated in this study were p53 and p63. For years, mutations in the p53 gene or alterations in its product have been correlated with poorer clinical outcomes in patients with bladder carcinoma,^(14)^ and to the presence of the encoded HPV oncoprotein, E6. In our study, no p53 associations were identified. The high rate of p53-positive tumors in our study suggests that this protein is a determinant factor for the carcinogenesis of bladder SCC.

Regarding p63 expression, all samples showed positive immunoreactivity. It is noteworthy that there was extensive p63 positivity in SCC of the bladder, with no correlation to any clinicopathological features analyzed in our study. p63 plays a key role in epithelial development in various organs. Therefore, immunostaining for p63 is useful for head and neck SCCs and pulmonary squamous neoplasia detection.^(27,28)^ A strong positive association between HPV and p63 has been reported in cervical carcinomas. Moreover, p63 has been helpful in differentiating pure SCCs from other histological types, with a loss of expression in squamous neoplasia transitioning to columnar or undifferentiated neoplasia.^(29)^

The clinicopathological features of the participants in this study showed similar frequencies to those of another study.^(4)^Hematuria was the principal symptom observed in 75% of the cases, followed by hypertension (64.29%) and pelvic pain (57.14%). In addition, our algorithm classifier, presented in figure 2B, emphasizes the importance of these clinical data for determining the degree of tumor differentiation in bladder SCC. This model identified hematuria and dysuria as important factors in determining the invasion level class, which was not initially relevant to the pathological classification of tumors. Moreover, our second ML model was also able to identify clinical parameters that were most informative in relation to the degree of tumor differentiation. Figure 2A highlights HPV infection status, lymphovascular invasion, gender, age, and compromised lymph nodes as important characteristics in this context. To ensure the generalizbility of the ML model, the LOOCV method was used. Although this approach is more appropriate for small datasets, some cases were not hit before neither after the validation method. We suggest that the low variance between the random training datasets and validation dataset may have defined this bias. The main strength of this study was to highlight the need to detach findings of statistical significance in associational measures from those of variable importance in tumor classification. The development of this ML approach does not require a large amount of data, which is particularly well suited when dealing with medical information. It is possible to understand the reasons behind the choices made by the algorithm, whcih can also deal with both categorical and numerical data. However, despite several ML research efforts in predicting the outcomes of patients with cancer, usage rates of such models remain low in clinical practice.

## CONCLUSION

HPV and p16 expression are not present in most non-schistosomiasis-related squamous cell carcinoma of the bladder at different stages of tumor invasion. These markers provide a benchmark for predicting the level of invasion using an machine learning model. Finally if deployed correctly, machine learning models can transform clinical and pathological data into accurate outcome predictions.
